# Dependence of spatial filtering by spike timing dependent synaptic plasticity on learning window

**DOI:** 10.1186/1471-2202-12-S1-P113

**Published:** 2011-07-18

**Authors:** Kazuhisa Fujita

**Affiliations:** 1 Department of Computer and Information Engineering, Tsuyama National College of Technology, Japan

## 

Spike timing dependent synaptic plasticity (STDP) plays an important role in temporal information processing. Whereas our result of the previous study showed that an interconnected network with STDP plays a role in spatial information processing [1]. The role is spatial filtering. In the present study, we studied the effect of learing window of STDP on spatial filtering by the interconnected network with STDP using computer simulation.

STDP learning is domated by learning window. Various types of learning window of STDP are found in various brain. In a hippocampus, a post-before-presynaptic spike pair couses synaptic efficacy to strengthen and a pre-before-postsynaptic spike pair courses synaptic efficacy to weaken (here we called this type learning “hippocampal type learning”). In a electrosensory lobe of an electric fish, the learning of STDP is reverse of hippocampal type learning (here we called “electric fish type learning”).

Using the learning window of hippocampal type, the network acted as spatial high-contrast filtering. Left figure of Fig.3 shows input image. Neuron on white and grey area receives high and low magnitude of input current, respectively. Middle figure in Fig.3 shows output image from the network. The output image means firing count of neurons of the network from 9000msec to 10000msec. In this case, firing count of neurons which received lower and higher input is lower and larger, respectively. Thus the network with STDP functioned as spatial high contrast filtering using learning window of hippocampal type .

Using the learning window of electric fish type, the network acted as spatial low-pass filter. Left figure of Fig.3 shows input image. Right figure of Fig.3 shows output image from the network. The output image means firing count of neurons of the network from 9000msec to 10000msec. In this case, spatial high frequency component was reduced and spatial low frequency component is enhanced in output. Thus the network functioned as spatial low-pass filter using learning window of electric fish type.

**Figure 1:**
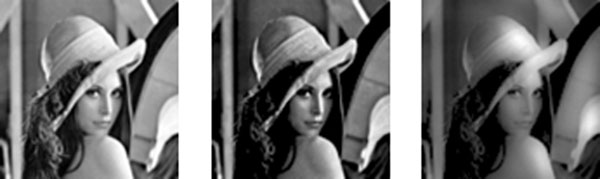
Left figure is an input image. Middle figure is output of the network with STDP applied hippocampal type learning window. Right figure is output of the network with STDP applied electric fish type learning window.

A function of spatial filtering was provided by an interconnected network with STDP. The function changed according to a type of learning window of STDP. In the preset study, we proposed two type of spatial filtering using two type of learning window. The spatial filtering was achieved by dynamically changing connection of the network based on STDP. The results of this study suggested that the interconnected network with STDP might provide appropriate spatial filtering for an optional image from changing network connection according to the image.
